# Supplementary material for: Lung tropism in hospitalized patients following infection with SARS-CoV-2 variants from D614G to Omicron BA.2
Source: Commun Med (Lond). 2023 Feb 25;3:32. doi: 10.1038/s43856-023-00261-5 (PMC9959956; doi:10.1038/s43856-023-00261-5)
Supplement: Supplementary file 2 — Supplementary Information [file 43856_2023_261_MOESM2_ESM.pdf]

Lung tropism in hospitalized patients following infection with SARS-CoV-2 variants from D614G to Omicron BA.2

Yosuke Hirotsu[1*¶], Yumiko Kakizaki[2¶], Akitoshi Saitoh[3], Toshiharu Tsutsui[2], Syunya Hanawa[2], Haruna Yamaki[2], Syuichiro Ide[2], Makoto Kawaguchi[2], Hiroaki Kobayashi[2], Yoshihiro Miyashita[2], and Masao Omata[4,5]

[1]Genome Analysis Center, Yamanashi Central Hospital, 1-1-1 Fujimi, Kofu, Yamanashi, Japan, [2]Lung Cancer and Respiratory Disease Center, Yamanashi Central Hospital, 1-1-1 Fujimi, Kofu, Yamanashi, Japan, [3]Department of Radiology, Yamanashi Central Hospital, 1-1-1 Fujimi, Kofu, Yamanashi, Japan, [4]Department of Gastroenterology, Yamanashi Central Hospital, 1-1-1 Fujimi, Kofu, Yamanashi, Japan, and [5]The University of Tokyo, 7-3-1 Hongo, Bunkyo-ku, Tokyo, Japan

***Corresponding author**: Yosuke Hirotsu, Genome Analysis Center, Yamanashi Central Hospital, Kofu, Japan

Email: hirotsu-bdyu@ych.pref.yamanashi.jp

Supplementary Information

- Supplementary Figure 1. CT scores in each lung lobe.
- Supplementary Figure 2. Comparison between time from most recent vaccination to infection and antibody titers, CT score, and clinical data.
- Supplementary Figure 3. Vaccination history and CT scores in BA.2-infected patients.
- Supplementary Table 1. Multiple comparisons of variants for presence/absence of pneumonia
- Supplementary Table 2. Treatments by vaccination history and COVID-19 pneumonia

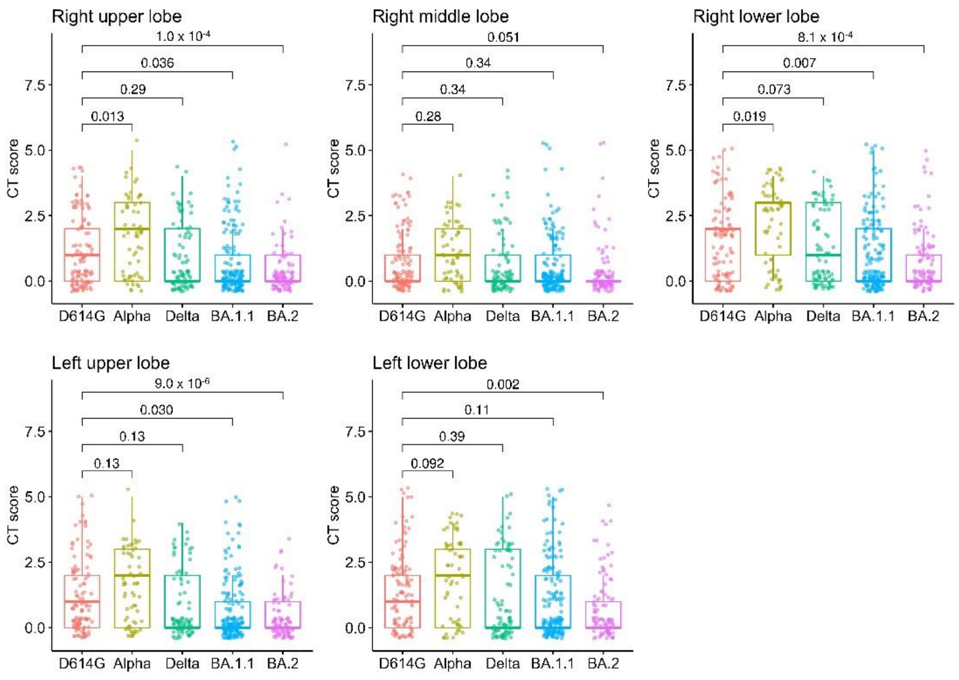

Supplementary Figure 1. CT scores in each lung lobe.

The boxplots show the CT score by lung lobe for each variant. We evaluated five lung lobes (right upper lobe, right middle lobe, right lower lobe, left upper lobe, and left lower lobe). Each box indicates the interquartile range (top: the third quartile; bottom: the first quartile) with a horizontal line indicating the median. Statistical analysis was performed by a t-test, and *P* values were adjusted for multiple comparisons.

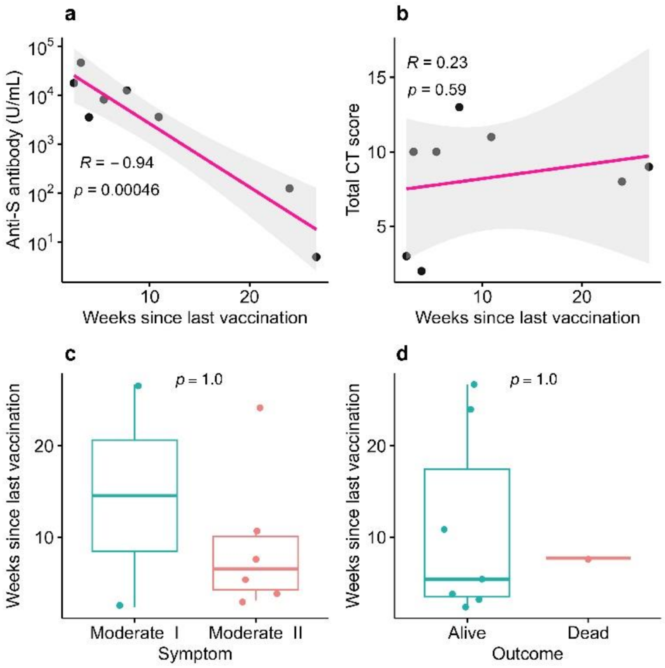

Supplementary Figure 2. Comparison between time from most recent vaccination to infection and antibody titers, CT score, and clinical data.

The data are from patients with a BA.2 breakthrough infection who had pneumonia. **a,b**) Correlation distributions of anti-S antibody titer (a) and total CT score (b) with time from the most recent vaccination to infection. The gray background indicates the 95% CI. The correlation coefficient (*R*) is noted. **c,d**) Boxplots showing the relationships between the time from the most recent vaccination to infection and patient symptoms (c), and outcomes (d). Each box indicates the interquartile range (top: the third quartile; bottom: the first quartile) with a horizontal line indicating the median. The statistical analysis was performed using the Mann-Whitney U test.

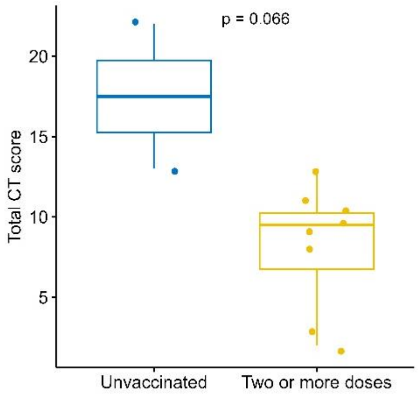

Supplementary Figure 3. Vaccination history and CT scores in BA.2-infected patients. Boxplots show the total CT scores of unvaccinated and vaccinated (two or more doses) BA.2-infected patients. Each box indicates the interquartile range (top: the third quartile; bottom: the first quartile) with a horizontal line indicating the median. The statistical analysis was performed using the Mann-Whitney U test.

Supplementary Table 1. Multiple comparisons of variants for presence/absence of pneumonia

Group1	Group2	n	p-value	adjusted p-value	Significance of adjusted p-value
D614G	Alpha	175	0.12	0.40	ns
D614G	Delta	198	0.31	0.40	ns
D614G	BA.1.1	277	4.3×10^{-7}	2.6×10^{-6}	****
D614G	BA.2	206	2.1×10^{-12}	1.9×10^{-11}	****
Alpha	Delta	145	0.029	0.087	ns
Alpha	BA.1.1	224	3.3×10^{-8}	2.3×10^{-7}	****
Alpha	BA.2	153	4.9×10^{-13}	4.9×10^{-12}	****
Delta	BA.1.1	247	6.3×10^{-4}	3.1×10^{-3}	**
Delta	BA.2	176	2.3×10^{-8}	1.8×10^{-7}	****
BA.1.1	BA.2	255	3.6×10^{-3}	0.014	*

ns: p > 0.05, *: p <= 0.05, **: p <= 0.01, ***: p <= 0.001, ****: p <= 0.0001

Supplementary Table 2. Treatments by vaccination history and COVID-19 pneumonia

	Unvaccinated, n = 256			Two or more doses, n = 221			Unknown, n = 37		
**Characteristic**	**n**	**Without, n = 114**	**With, n = 142**	**n**	**Without, n = 183**	**With, n = 38**	**n**	**Without, n = 17**	**With, n = 20**
**Age,** median (IQR)	256	43 (28, 68)	59 (45, 76)	221	73 (58, 88)	80 (71, 86)	37	60 (56, 77)	70 (60, 83)
**Sex**, n (%)	256			221			37		
Female		51 (45%)	49 (35%)		93 (51%)	10 (26%)		8 (47%)	8 (40%)
Male		63 (55%)	93 (65%)		90 (49%)	28 (74%)		9 (53%)	12 (60%)
**Remdesivir**, n (%)	256	33 (29%)	91 (64%)	221	78 (43%)	29 (76%)	37	9 (53%)	18 (90%)
**Casirivimab and imdevimab**, n (%)	256	4 (3.5%)	4 (2.8%)	221	6 (3.3%)	1 (2.6%)	37	0 (0%)	0 (0%)
**Sotrovimab**, n (%)	256	20 (18%)	2 (1.4%)	221	7 (3.8%)	2 (5.3%)	37	0 (0%)	0 (0%)
**Nirmatrelvir and Ritonavir**, n (%)	256	2 (1.8%)	0 (0%)	221	5 (2.7%)	0 (0%)	37	0 (0%)	0 (0%)
**Molnupiravir**, n (%)	256	0 (0%)	0 (0%)	221	57 (31%)	4 (11%)	37	0 (0%)	0 (0%)
**Tocilizumab**, n (%)	256	0 (0%)	10 (7.0%)	221	0 (0%)	4 (11%)	37	0 (0%)	3 (15%)
**Baricitinib**, n (%)	256	1 (0.9%)	25 (18%)	221	1 (0.5%)	4 (11%)	37	1 (5.9%)	6 (30%)
**Dexamethasone**, n (%)	256	8 (7.0%)	91 (64%)	221	11 (6.0%)	20 (53%)	37	2 (12%)	15 (75%)
**Prednisolone**, n (%)	256	0 (0%)	5 (3.5%)	221	0 (0%)	0 (0%)	37	0 (0%)	0 (0%)

Without, No evidence of typical COVID-19 pneumonia by CT; With, Evidence of typical COVID-19 pneumonia by CT; IQR, interquartile range
