# Supplementary material for: Lung tropism in hospitalized patients following infection with SARS-CoV-2 variants from D614G to Omicron BA.2
Source: Commun Med (Lond). 2023 Feb 25;3:32. doi: 10.1038/s43856-023-00261-5 (PMC9959956; doi:10.1038/s43856-023-00261-5)
Supplement: Supplementary file 3 — Description of Additional Supplementary Files [file 43856_2023_261_MOESM3_ESM.pdf]

Description of Additional Supplementary Files

File Name: Supplementary Data 1
Description: Patient characteristics and presence of pneumonia on CT images

File Name: Supplementary Data 2
Description: Patient characteristics of BA.2 infected patients

File Name: Supplementary Data 3
Description: Source data for the Figures and Tables
