# Supplementary material for: Lung tropism in hospitalized patients following infection with SARS-CoV-2 variants from D614G to Omicron BA.2
Source: Commun Med (Lond). 2023 Feb 25;3:32. doi: 10.1038/s43856-023-00261-5 (PMC9959956; doi:10.1038/s43856-023-00261-5)
Supplement: Supplementary file 7 — Reporting Summary [file 43856_2023_261_MOESM7_ESM.pdf]

nature portfolio

Corresponding author(s): Yosuke Hirotsu

Last updated by author(s): Jan 10, 2023

Reporting Summary

Nature Portfolio wishes to improve the reproducibility of the work that we publish. This form provides structure for consistency and transparency in reporting. For further information on Nature Portfolio policies, see our Editorial Policies and the Editorial Policy Checklist.

Statistics

For all statistical analyses, confirm that the following items are present in the figure legend, table legend, main text, or Methods section.

n/a	Confirmed	
☐	☒	The exact sample size (*n*) for each experimental group/condition, given as a discrete number and unit of measurement
☒	☐	A statement on whether measurements were taken from distinct samples or whether the same sample was measured repeatedly
☐	☒	The statistical test(s) used AND whether they are one- or two-sided<br>*Only common tests should be described solely by name; describe more complex techniques in the Methods section.*
☒	☐	A description of all covariates tested
☐	☒	A description of any assumptions or corrections, such as tests of normality and adjustment for multiple comparisons
☐	☒	A full description of the statistical parameters including central tendency (e.g. means) or other basic estimates (e.g. regression coefficient) AND variation (e.g. standard deviation) or associated estimates of uncertainty (e.g. confidence intervals)
☐	☒	For null hypothesis testing, the test statistic (e.g. *F*, *t*, *r*) with confidence intervals, effect sizes, degrees of freedom and *P* value noted<br>*Give P values as exact values whenever suitable.*
☒	☐	For Bayesian analysis, information on the choice of priors and Markov chain Monte Carlo settings
☒	☐	For hierarchical and complex designs, identification of the appropriate level for tests and full reporting of outcomes
☐	☒	Estimates of effect sizes (e.g. Cohen's *d*, Pearson's *r*), indicating how they were calculated

Our web collection on statistics for biologists contains articles on many of the points above.

Software and code

Policy information about availability of computer code

Data collection	All data was collected with the StepOnePlus Real-Time PCR, the FilmArray Torch system, and the GeneXpert for SARS-CoV-2 detection, the Ion Torrent Genexus System for genome sequencing, the LUMIPULSE G600II for antigen test, and the cobas® 8000 automated platform for serological analysis. Data on the medical condition, vaccination status, and disease course were collected from patient interviews and retrospectively examined the electronic medical records. CT images were obtained with the Aquilion 64 or the Aquilion 64CX system.
Data analysis	All statistical tests and visualizations were performed with R, version 4.1.1 (R Foundation for Statistical Computing) or RStudio (https://www.rstudio.com/). The following R packages were used for data cleaning, analysis, and visualization: ggplot2 (v3.3.5), dplyr (v1.0.7), tidyr (v1.1.3), patchwork (v1.1.1), gtsummary (v1.5.2), and flextable (v.0.7.0). A multivariate logistic regression analysis was performed to evaluate the effects of age (over or under 65 years), sex, viral lineage, and vaccination status on the likelihood of having COVID-19 pneumonia. Odds ratios (ORs) and the corresponding 95% confidence intervals (CIs) were calculated. P values <0.05 were considered to be statistically significant.

For manuscripts utilizing custom algorithms or software that are central to the research but not yet described in published literature, software must be made available to editors and reviewers. We strongly encourage code deposition in a community repository (e.g. GitHub). See the Nature Portfolio guidelines for submitting code & software for further information.

Data

Policy information about availability of data

All manuscripts must include a data availability statement. This statement should provide the following information, where applicable:

- Accession codes, unique identifiers, or web links for publicly available datasets
- A description of any restrictions on data availability
- For clinical datasets or third party data, please ensure that the statement adheres to our policy

The sequences of SARS-CoV-2 genomes are available on GISAID (www.gisaid.org). The source data underlying Figures and Tables is available in the Supplementary Table 4 and Supplementary Data file.

Human research participants

Policy information about studies involving human research participants and Sex and Gender in Research.

Reporting on sex and gender	Data on self-report sex were retrospectively examined the electronic medical records.
Population characteristics	See above.
Recruitment	Except for patients who do not meet participation requirements, all hospitalized participants were included in this study. There aren't any potential self-selection bias.
Ethics oversight	The Institutional Review Board of the Clinical Research and Genome Research Committee at our hospital approved this study and the use of an opt-out consent method (approval no. C2019-30).

Note that full information on the approval of the study protocol must also be provided in the manuscript.

Field-specific reporting

Please select the one below that is the best fit for your research. If you are not sure, read the appropriate sections before making your selection.

☒ Life sciences ☐ Behavioural & social sciences ☐ Ecological, evolutionary & environmental sciences

For a reference copy of the document with all sections, see nature.com/documents/nr-reporting-summary-flat.pdf

Life sciences study design

All studies must disclose on these points even when the disclosure is negative.

Sample size	All enrolled participants were hospitalized at Yamanashi Central Hospital between February 2020 and August 2022, and were infected with SARS-CoV-2 confirmed by RT-qPCR assay or antigen test using by nasopharyngeal swabs. A total of 514 patients who had undergone CT scanning were eligible in this study.
Data exclusions	We excluded 84 hospitalized patients who did not undergo SARS-CoV-2 genomic analysis due to low viral loads. We also excluded 21 individuals who initially tested positive for SARS-CoV-2 by PCR but were likely false positives according to subsequent analyses. A further 110 patients, including children or pregnant women who had not undergone CT scanning, were also excluded. Only one patient with the Gamma variant was detected during this period, but this case was removed from subsequent analyses because of the small number of Gamma variant detections.
Replication	N/A
Randomization	N/A
Blinding	N/A

Reporting for specific materials, systems and methods

We require information from authors about some types of materials, experimental systems and methods used in many studies. Here, indicate whether each material, system or method listed is relevant to your study. If you are not sure if a list item applies to your research, read the appropriate section before selecting a response.

Materials & experimental systems

n/a	Involved in the study
☒	☐ Antibodies
☒	☐ Eukaryotic cell lines
☒	☐ Palaeontology and archaeology
☒	☐ Animals and other organisms
☒	☐ Clinical data
☒	☐ Dual use research of concern

Methods

n/a	Involved in the study
☒	☐ ChIP-seq
☒	☐ Flow cytometry
☒	☐ MRI-based neuroimaging
